# Mortality burden due to liver cirrhosis and hepatocellular carcinoma in Ghana; prevalence of risk factors and predictors of poor in-hospital survival

**DOI:** 10.1371/journal.pone.0274544

**Published:** 2022-09-13

**Authors:** Yvonne A. Nartey, Samuel O. Antwi, Ansumana S. Bockarie, Lindsey Hiebert, Henry Njuguna, John W. Ward, Yaw A. Awuku, Amelie Plymoth, Lewis R. Roberts

**Affiliations:** 1 Department of Medical Epidemiology and Biostatistics, Karolinska Institute, Stockholm, Sweden; 2 Department of Internal Medicine, Cape Coast Teaching Hospital, Cape Coast, Ghana; 3 Division of Epidemiology, Department of Health Sciences Research, Mayo Clinic, Jacksonville, Florida, United States of America; 4 Department of Internal Medicine, University of Cape Coast School of Medical Sciences, Cape Coast, Ghana; 5 Coalition for Global Hepatitis Elimination, Task Force for Global Health, Decatur, GA, United States of America; 6 Department of Internal Medicine, University of Health and Allied Sciences, Hohoe, Ghana; 7 Department of Medicine, Division of Gastroenterology and Hepatology, Mayo Clinic, Rochester, Minnesota, United States of America; Non-Communicable Diseases Research Center, Endocrinology and Metabolism Population Sciences Institute, Tehran University of Medical Sciences, ISLAMIC REPUBLIC OF IRAN

## Abstract

Liver-related diseases, including liver cirrhosis and hepatocellular carcinoma (HCC), are significant causes of mortality globally. Specific causes and predictors of liver-related mortality in low resource settings require assessment to help inform clinical decision making and develop strategies for improved survival. The objectives of this study were to determine the proportion of liver-related deaths associated with liver cirrhosis, HCC, and their known risk factors, and secondly to determine predictors of in-hospital mortality among cirrhosis and HCC patients in Ghana. We first performed a cross-sectional review of death register entries from 11 referral hospitals in Ghana to determine the proportion of liver-related deaths and the proportion of risk factors associated with these deaths. Secondly, we conducted a retrospective cohort review of 172 in-patient liver cirrhosis and HCC cases admitted to a tertiary referral centre and determined predictors of in-hospital mortality using binary logistic regression and Kaplan-Meier survival analysis. In total, 8.8% of deaths in Ghanaian adults were due to liver-related causes. The proportion of liver-related deaths attributed to HBV infection was 48.8% (95% CI: 45.95–51.76), HCV infection was 7.0% (95% CI: 5.58–8.45), HBV-HCV co-infection 0.5% (95% CI: 0.1–0.9) and alcohol was 10.0% (95% CI: 8.30–11.67). Of 172 cases of HCC and liver cirrhosis, the in-patient mortality rate was 54.1%. Predictors of in-patient mortality in cirrhotic patients were increasing WBC (OR = 1.14 95% CI: 1.00–1.30) and the revised model for end-stage liver disease with sodium (MELD-Na) score (OR = 1.24 95% CI: 1.01–1.54). For HCC patients, female sex (OR = 3.74 95% CI: 1.09–12.81) and hepatic encephalopathy (grade 1) were associated with higher mortality (OR = 5.66 95% CI: 1.10–29.2). In conclusion, HBV is linked to a high proportion of HCC-related deaths in Ghana, with high in-hospital mortality rates that require targeted policies to improve survival.

## Introduction

Liver-related diseases, including chronic viral hepatitis, liver cirrhosis and hepatocellular carcinoma (HCC), remain a significant cause of morbidity and mortality globally. HCC was the third leading cause of cancer-related death in 2020 globally [1]. In sub-Saharan Africa, HCC is the second most common cause of cancer death in males and fourth in females [2]. The age-standardised mortality rate for liver-related mortality is highest in sub-Saharan Africa [3], where the median survival for HCC is estimated to be 10.9 months in Egypt and 2.5 months in other African countries [4]. Ghana has the fourth highest age-standardised incidence rate for HCC in Africa at 16.9 per 100,000 population [5]. Cirrhosis is commonly a predetermining step in HCC development, however even in the absence of HCC, it is independently responsible for significant morbidity and mortality. In 2019, 1.47 million deaths worldwide were attributed to liver cirrhosis [6] and in the same year, accounted for 8.44 million Disability Adjusted Life Years (DALYs) [7]. In Ghana, cirrhosis was ranked the 10^th^ highest cause of mortality in 2019, with an estimated increase of 12.3% in total number of deaths due to cirrhosis between 2009 and 2019 [8]. There is evidence that liver-related mortality has been under-reported even in highly resourced countries [9], therefore, it is probable that the true burden of mortality in Africa is much higher than the current estimates. It is therefore necessary to undertake studies to provide improved evidence of the liver disease burden in low- and middle-income settings.

Liver-related mortality may occur due to clinical complications of liver cirrhosis and HCC, such as hepatic encephalopathy, upper gastrointestinal bleeding and hepatorenal syndrome [10]. Mortality may also be related to patient demographics, including age at diagnosis, clinical characteristics, such as the presence of co-morbidities, and stage of disease [11]. Furthermore, the aetiology or risk factors associated with cirrhosis and HCC, may increase the mortality rate [11]. Among hospitalised patients with end-stage liver disease, predictors of mortality include elevated white blood cell (WBC) count, sepsis, increasing MELD score and presence of shock [12, 13]. In Ghana, Duah et al identified hepatic encephalopathy, elevated creatinine, international normalized ratio (INR) and the revised model for end-stage liver disease with sodium (MELD-Na) score as predictors of mortality in hospitalised cirrhosis patients [14]. An assessment of predictors of mortality in higher level centres in Ghana which are better equipped and often serve as the final point of referral for end-stage liver disease (ESLD) cases is warranted to better understand mortality patterns associated with ESLD. Furthermore, there is a paucity of studies on predictors of mortality in HCC patients in Ghana, although the literature shows that patients often present at advanced stages of the disease [15, 16]. An improved understanding is important to help inform clinical decision making, direct policy and develop preventive strategies for improved survival.

The aim of this study was to determine the proportion of liver-related deaths attributable to liver cirrhosis and hepatocellular carcinoma in Ghana, and to determine the proportional prevalence of common risk factors such as HBV infection associated with liver cirrhosis, liver cancer and chronic liver disease mortality. Finally, we aimed to determine clinical factors associated with poor survival for liver cirrhosis and HCC patients admitted to a tertiary referral centre in Ghana.

## Materials and methods

### Study design and study sites

In this two-part study, we first performed a retrospective review of medical certification of cause of death (MCCD) registers in 11 referral hospitals in Ghana comprising district, regional and teaching hospitals to determine the proportion of liver-related deaths attributable to liver cirrhosis, hepatocellular carcinoma, and chronic liver disease.

These hospitals were purposively selected after zoning the country into upper, middle, and lower zones to obtain geographically representative hospitals. In each zone, at least one district, one regional, and where available one teaching hospital was selected, to represent the levels of healthcare in the Ghana Health Service. In total, 1214 MCCD certificates signed by practitioners between January 2018 to December 2020 were extracted for review from 4 district, 4 regional, 2 teaching hospitals and 1 faith-based institution.

MCCD registers in Ghana use the standard WHO format for certification of medical cause of death [17]. Specifically, there is a first part (Part I) which describes the disease or condition leading to death and its antecedent causes, and a second part (Part II) which describes other significant conditions contributing to death. Within these registers, the underlying causes of death for liver-related mortality were listed as either HCC, liver cirrhosis, or in some cases chronic liver disease (CLD). To determine the proportion of all deaths assigned to liver-related causes between 2018–2020, we compared the number of liver-related deaths reported in MCCD registers between 2018–2020 with the total number of deaths reported in the District Health Information Management System (DHIMS2) of Ghana, which is a national database where health related data from Ghana Health Service facilities are reported. Seven out of the 11 sites for whom MCCD registers were reviewed, reported mortality data in the Ghana DHIMS2 database between 2018–2020. To determine risk factors associated with liver-related conditions, we further abstracted data on the antecedent cause of death including HBV, HCV, alcohol, and other known risk factors of liver-related mortalities in Ghana. For entries where no risk factor was stated, this was categorised as unspecified, and this category was included in the analysis of proportions. This work was part of the Hepatitis Evaluations to Amplify Testing (HEAT) Project, which was a nationwide cross-sectional study on viral hepatitis-related testing and treatment in Ghana. Mortality data collected for this study formed a small part of the larger HEAT study.

In the second part of the study, we selected one teaching hospital, the Cape Coast Teaching Hospital, a tertiary referral centre, to determine factors associated with liver-related mortality. As a teaching hospital, this facility receives referral cases from lower cadres of the health system and was thus purposively selected for this reason. Furthermore, availability of electronic health records for the entirety of the study period was an additional reason for selection. At the Cape Coast Teaching Hospital, we performed a retrospective cohort study of end-stage liver disease patients admitted between January 2018 to December 2021.

### Data collection

Data collection took place between January and December 2021. In total, 1214 MCCD register entries were extracted in the first part of this study. A data extraction form was used to obtain summary data on the underlying cause of death and associated risk factors related to mortality.

For the second part of the study 172 liver cirrhosis and HCC cases admitted at the Cape Coast Teaching Hospital were reviewed. Data were retrieved from electronic health information management system records. Diagnosis was based on clinical history and examination, biochemical and radiological evidence of liver cirrhosis or liver cancer as recorded in the medical records. No histopathological diagnosis was performed for any of the cases reviewed. For in-patient data, where there was the presence of HCC on a background of cirrhosis, the data were categorized as HCC. Additionally, where one individual had two or more admissions, each admission was counted as an individual case for analysis. Exclusion criteria were pregnancy, and admission for less than one day for procedures such as therapeutic paracentesis.

Data validation methods included the use of template data collection instruments designed for the HEAT project. These instruments were evaluated during a stakeholder engagement meeting to ensure that the correct type and range of data were to be collected. Following consensus agreement of data collection tools, research assistants were trained on the use of the tools with one site serving as a pilot site. Response questions for data entry in Microsoft Excel were restricted to finite responses and drop-down lists where possible to limit aberrant responses. Maximum and minimum entry limits were set to limit entry of incorrect data ranges. Missing entries were reviewed to determine if entries were omitted in error or legitimately missing from records. Following data-collection, presentation of the field data was presented in a second stakeholder meeting for evaluation and review.

### Ethical approval

The study was approved by the ethical review committees of the Ghana Health Service (ERC number GHS-ERC 002/10/20), Korle Bu Teaching Hospital (KBTH-STC 00083/2021), Komfo-Anokye Teaching Hospital (KATH IRB/AP/099/21) and the Cape Coast Teaching Hospital (CCTHERC/EC/2021/117). All data collected was secondary data from review of archived medical records and were de-identified and anonymised prior to analysis. Informed consent was not required and was waived by all ethical review committees.

### Statistical analysis

Differences in the distribution of participant characteristics were examined using means with standard deviation (SD) or median with interquartile range (IQR) for continuous variables, and frequencies (percentages) for categorical variables. Tests for differences between groups were performed using Student’s t-test for continuous variables and chi-square tests for categorical variables. Proportions of risk factors associated with HCC diagnosis or liver-related deaths were determined using percentages with corresponding 95% confidence intervals (CIs). Predictors of in-hospital mortality were determined using binary logistic regression adjusted for age, sex, and risk factors to calculate odds ratios and 95% CIs. In the logistic regression models, HCC and death were modelled separately as outcomes, and clinical variables modelled as predictors included age (continuous), sex (male/female), WBC count (continuous), INR (continuous), platelet count (continuous), total bilirubin (continuous) MELD-Na (continuous), hepatic encephalopathy (present/absent) and upper GI bleeding (present/absent). The decision to keep a variable in the model was based on clinical and statistical significance. For statistical significance, a significance level of 0.05 was used. Additionally, we performed survival analyses using the Kaplan-Meier methods comparing survival probabilities between cirrhosis and HCC, survival probabilities by MELD-Na score (< = 19, 20–29, >30) and Child-Pugh score (A, B, C). For these analyses, the Kaplan-Meier log-rank test was used to determined differences in survival probabilities between groups. All statistical tests were two-sided and a p-value less than 0.05 was considered statistically significant. The statistical analyses were performed using Stata, version 17.0; StataCorp software.

## Results

### Mortality registers and liver related mortality

**Fig 1:**
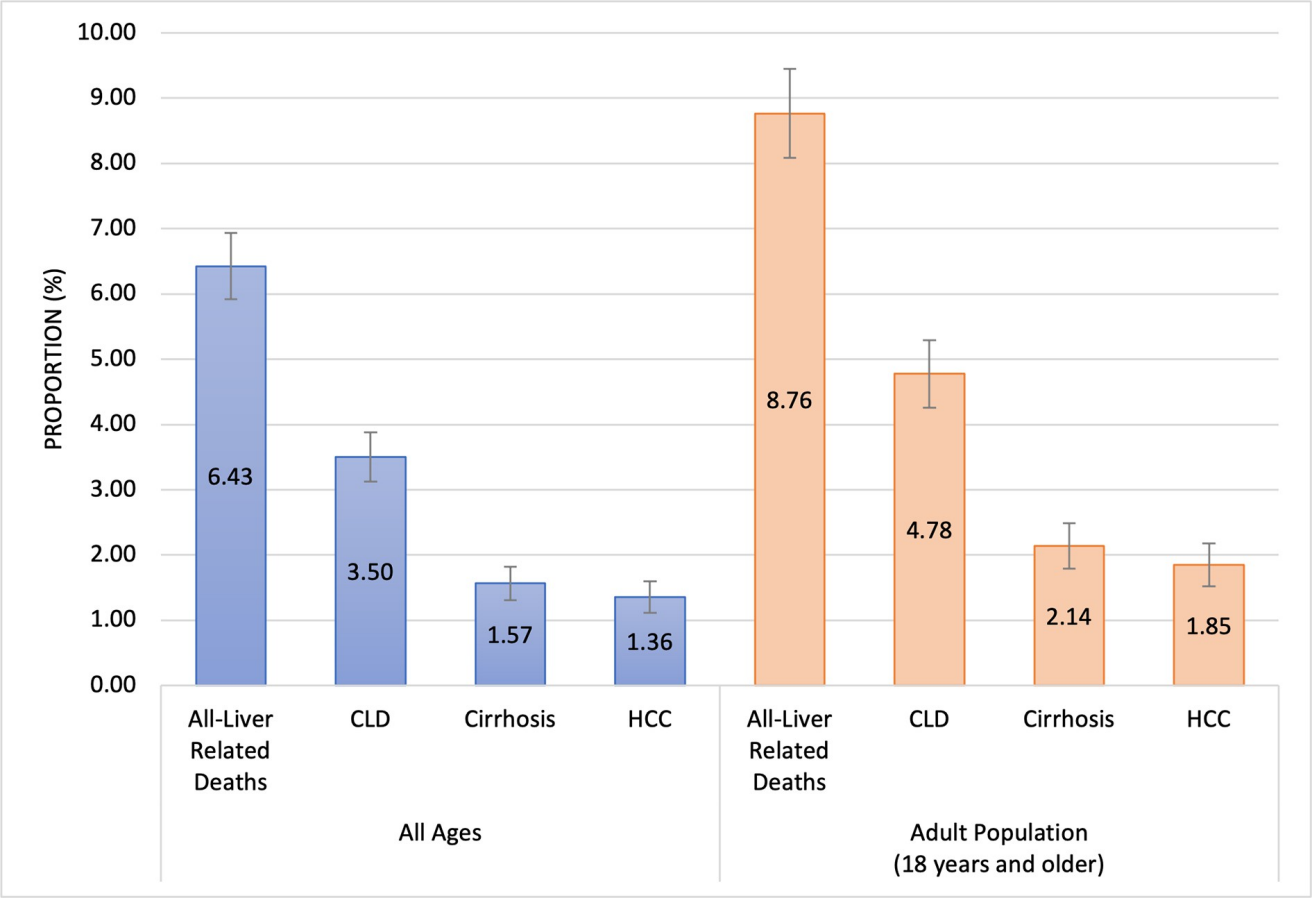
Proportion of risk factors associated with deaths from liver-related diseases reported in the Ghana District Health Information Management System (DHIMS2) between 2018–2020 from seven referral centres in Ghana with 95% CI. Abbreviations: CLD- Chronic Liver Disease, HCC- Hepatocellular Carcinoma.

We assessed the total number of deaths reported in the DHIMS2 from referral sites for all ages and for the adult population (18 years and above) to determine the proportion of all deaths assigned to liver-related causes between 2018–2020. Out of 8995 reported deaths from all causes for all ages, 578 (6.4%, 95% CI: 5.9–6.9) were due to liver-related diseases (**Fig 1**). For the adult population, 8.8% (95% CI: 8.1–9.5) of deaths were liver-related. Of these, 1.9% (95% CI: 1.5–2.2) were due to HCC and 2.1% (95% CI: 1.8–2.5) were due to liver cirrhosis.

**Fig 2:**
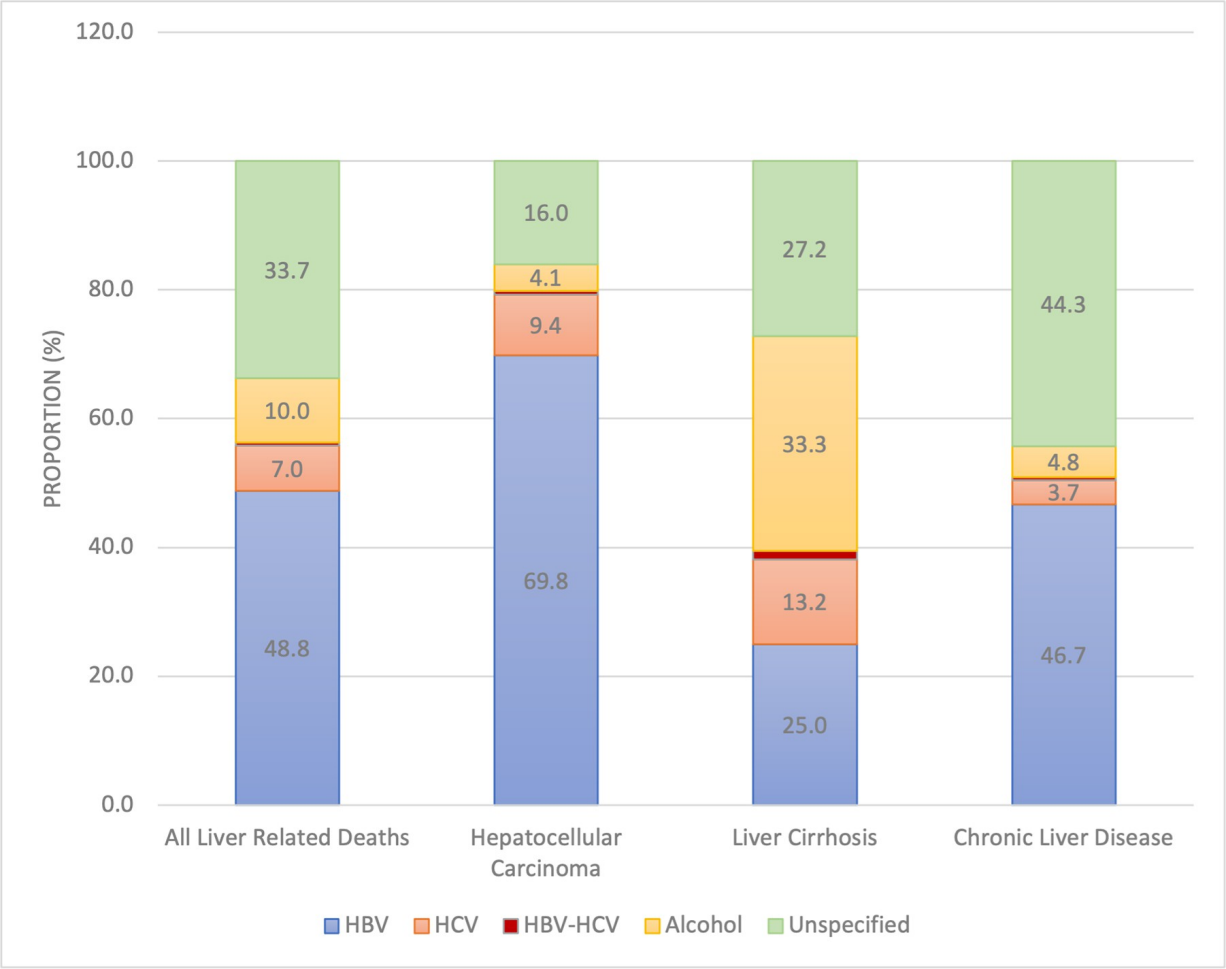
Proportion of risk factors associated with death from hepatocellular carcinoma, liver cirrhosis, and chronic liver disease in 11 referral hospitals in Ghana. Abbreviations: HBV- Hepatitis B Virus, HCV- Hepatitis C Virus.

Out of all liver-related deaths certified in the MCCD registers, 318 (26.2%) deaths from HCC, 228 (18.8%) from liver cirrhosis, and 668 (55.0%) from chronic liver disease, were reviewed for proportional prevalence of various known risk factors for liver disease. For all liver-related deaths, the prevalence of HBV infection was 48.8% (95% CI: 46.0–51.8), HCV infection was 7.0% (95% CI: 5.6–8.5), HBV-HCV co-infection 0.5% (95% CI: 0.1–0.9) and alcohol was 10.0% (95% CI: 8.3–11.7) (**Fig 2**). A higher proportion of HCC deaths was associated with HBV infection (69.8% in HCC vs. 25% in cirrhotic patients, p < 0.001), whilst a higher proportion of deaths from liver cirrhosis was associated with alcohol use (33.3% in cirrhotic vs. 25.0% in HCC patients p<0.001) (**Fig 2**).

Approximately half of liver-related deaths were certified by practitioners as unspecified chronic liver disease; for these deaths, HBV infection was the most predominant risk factor. Furthermore, for approximately one third of all liver-related mortalities recorded, no associated risk factor was specified. Missing or unspecified cause of death was most common for deaths certified as chronic liver disease compared with cirrhosis and HCC (44.3% for CLD vs 27.2% for cirrhosis vs. 16% for HCC).

### Factors associated with in-patient mortality in a tertiary referral centre

#### Baseline characteristics

The medical records of 172 patients with liver cirrhosis and liver cancer were reviewed to determine clinical factors associated with in-hospital mortality. Of these, there were 96 (55.8%) cirrhosis cases and 76 (44.2%) HCC cases. **Table 1** summarises the baseline characteristics of patients included in the study. The cirrhosis and HCC patients did not differ by age, sex, presence of comorbidities, referral status, or duration of admission. However, haemoglobin, platelet count, serum albumin and alpha-fetoprotein (AFP) levels were significantly higher in the HCC patients than the cirrhosis patients. No other differences were noted (**Table 1**).

**Table 1:** Baseline characteristics of liver cirrhosis and HCC patients.

	All patients	Cirrhosis	HCC	p value
	n = 172	n = 96	n = 76	
Age in years, mean ± SD	42 ±14.5	41.7 14.0	42.8 15.4	0.63
Gender				
Male	112 (65.1)	62 (64.6)	50 (65.8)	0.87
Comorbidity				
Yes	12 (13.8)	5 (11.9)	7 (15.6)	0.62
Referral				
Yes	31 (36.5)	17 (40.5)	14 (32.5)	0.45
Duration of admission in days, median (IQR)	6 (4–11.5)	7 (4–11)	6 (3–12)	0.63
Haemoglobin g/dL median (IQR)	9.5 (7.7–11.9)	9.2 (7.3–11.1)	10.5 (8.7–13.1)	**0.004**
WBC (X10^9^/L) median (IQR)	8.4 (5.7–12.8)	7.9 (5.2–14.0)	9.0 (6.5–12.5)	0.40
Platelet (KU/L) median (IQR)	154 (85.5–266.5)	114 (66.5–199)	238 (158–327)	**<0.001**
Albumin (g/L) median (IQR)	28.4 (23.5–32.9)	25.3 (21.6–30)	32.3 (29–37.3)	**<0.001**
Total bilirubin (mg/dl) median (IQR)	41.9 (18.7–117.4)	35.7 (17.4–116.1)	56.0 (20.1–123.1)	0.26
AFP (ng/ml) median (IQR)	207.3 (17.6–15663)	23.3 (11.8–113.5)	301(105.5–30815)	**0.046**
INR median (IQR)	1.9 (1.6–2.6)	2.1 (1.6–3.0)	1.8 (1.6–2.2)	0.25
Child-Pugh Score				
A	18 (13.0)	9 (10.1)	9 (18.4)	0.10
B	53 (38.4)	31 (34.8)	22 (44.9)	
C	67 (48.6)	49 (55.1)	18 (36.7)	

Abbreviations: WBC = White blood cell AFP = Alpha fetoprotein INR = International normalised ratio

**Fig 3:**
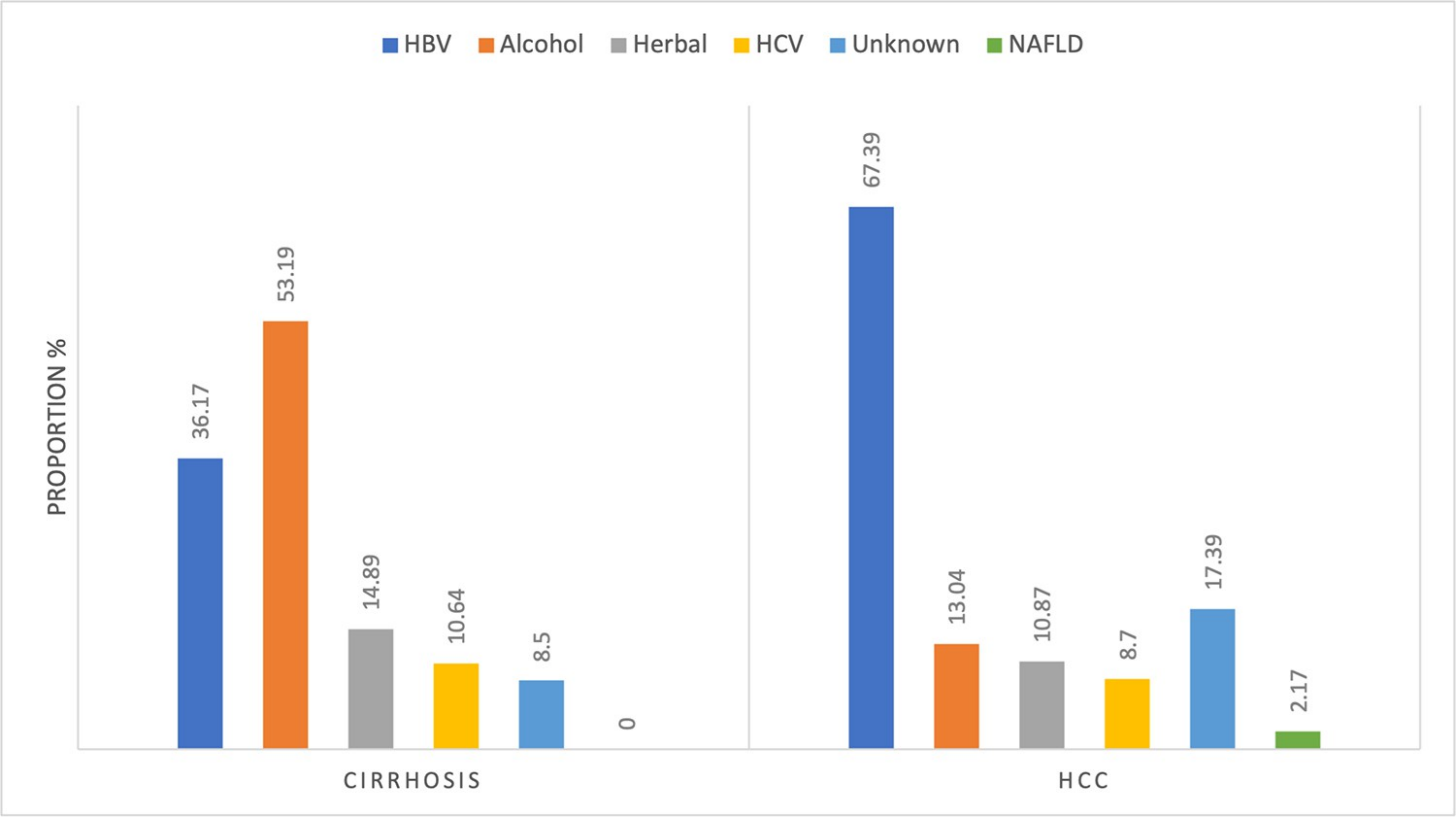
Proportion of risk factors for cirrhosis and HCC in patients seen at a tertiary referral centre in Ghana. Abbreviations: HBV- Hepatitis B Virus, HCV- Hepatitis C Virus, NAFLD- Non-alcoholic Fatty Liver Disease.

When proportions of risk factors for HCC and cirrhosis were assessed, we found that a higher proportion of HCC patients were infected with HBV compared with cirrhotic patients (67% vs. 36%, respectively) (**Fig 3**). In contrast, alcohol use was more common among cirrhotic patients than HCC patients (53% vs. 13%, respectively) (**Fig 3**).

On univariable logistic regression analysis with cirrhosis as the reference category, we found that patients admitted with HCC had a 2.7 times higher odds of HBV infection than those admitted with cirrhosis (OR = 2.73, 95% CI: 1.47–5.09) (**Table 2**). Further, HCC patients had a lower odds of having alcohol as a risk factor for their diagnosis (OR = 0.29, 95% CI: 0.15–0.58). Each one unit increase in haemoglobin level was associated with a higher odds of having HCC (OR = 1.15, 95% CI: 1.03–1.30) and each one unit increase in albumin level was associated with a higher odds of having HCC (OR = 1.20, 95% CI: 1.11–1.27) in the univariate analysis. Upon multivariable analysis with adjustment for age and sex, included risk factors and clinical variables (**Table 2**), the association between HBV infection and HCC remained significant (OR = 4.49, 95% CI: 1.13–17.89) as compared with cirrhosis, as did the association between HCC and albumin level (OR = 1.20 95% CI: 1.10–1.32). No other significant finding was observed in the multivariable model (**Table 2**).

**Table 2:** Logistic regression analysis of clinical variables associated with HCC (outcome) compared with cirrhosis in patients admitted to a tertiary referral centre.

	Univariable		Multivariable	
	OR (95% CI)	p value	Adjusted* OR	p-value
HBV	2.73 (1.47–5.09)	**<0.001**	**4.49 (1.13–17.89)**	**0.03**
HCV	1.28 (0.36–4.60)	0.70	3.35 (0.32–34.60)	0.31
Alcohol	0.29 (0.15–0.58)	**<0.001**	1.63 (0.43–6.09)	0.47
Herbal medication	1.30 (0.50–3.45)	0.60	0.70 (0.14–3.44)	0.66
Haemoglobin g/dL	1.15 (1.03–1.30)	**0.02**	0.98 (0.84–1.16)	0.85
Platelet (KU/L)	1.00 (1.00–1.01)	<0.001	1.00 (1.00–1.01)	0.04
Albumin (g/L)	1.20 (1.11–1.27)	**<0.001**	**1.20 (1.10–1.32)**	**<0.001**
INR	1.08 (0.92–1.25)	0.35		
AFP (ng/ml)	1.01 (0.99–1.03)	0.27		
WBC(X10^9^/L)	1.01 (0.96–1.05)	0.78		

Multivariable model adjusted for age (continuous) gender (male, female), HBV (yes, no), HCV (yes, no), Alcohol (yes, no), Herbal medication (yes, no), haemoglobin (continuous), platelet (continuous) and albumin (continuous). INR, AFP and WBC omitted from final model due to p>0.05 on univariable analysis. Abbreviations: AFP, alpha-fetoprotein; INR, international normalized ratio; HBV, hepatitis B virus, HCV, hepatitis C virus; WBC, white blood cells.

#### In-patient mortality

Of the 172 patients admitted, 95 patients (54%) died during admission, comprising 48.9% of cirrhosis and 60.5% of HCC patients. There was no statistically significant difference in the in-patient mortality rate between the HCC or cirrhosis patients (p = 0.13). **Table 3** shows the baseline characteristics of patients who died compared with those who survived separately for cirrhosis patients and HCC patients. Among cirrhosis patients, HCV infection was the only difference in the baseline characteristics of those who died during admission compared to those who were discharged (p = 0.02). Among the HCC patients, those who died during admission were more likely to be female as compared to those discharged (p = 0.01). Univariable logistic regression analysis of clinical variables associated with in-patient mortality for patients with cirrhosis included elevated WBC count, higher INR, higher total albumin, and higher MELD-Na score, Child-Pugh Score B or C, and the presence of hepatic encephalopathy (**Table 4**).

**Table 3:** Baseline characteristics of patients discharged vs. patients who died on admission by diagnosis.

Cirrhosis				
	All patients	Discharged	Died	p value
	n = 96	n = 49	n = 47	
Age in years, mean ±SD	41.7 ±13.9	41.0 ±13.4	42.6 ±14.6	0.57
Sex n, %				
Male	62 (64.6)	34 (69.4)	28 (59.6)	0.32
Female	34 (35.4)	15 (30.6)	19 (40.3)	
Attributable Factor				
HBV	37 (38.5)	20 (40.8)	17 (36.2)	0.64
HCV	5 (5.21)	0 (0)	5 (10.6)	**0.02**
Alcohol	44 (45.8)	19 (38.8)	25 (53.2)	0.16
Herbal medication	9 (9.4)	2 (4.1)	7 (14.9)	0.07
NAFLD	1 (1.0)	1 (2.0)	0	
Unknown	9 (9.4)	5 (10.2)	4 (8.5)	
Comorbidity				
Yes	5 (11.9)	3 (16.7)	2 (8.3)	0.41
No	37 (88.1)	15 (83.3)	22 (91.7)	
**HCC**				
	All patients	Discharged	Died	p value
	n = 76	n = 30	n = 46	
Age in years, mean ±SD	42.8 ±15.4	39.0 ±13.8	45.3 ±15.9	0.08
Sex n, %				
Male	50 (65.8)	25 (83.3)	25 (54.35)	**0.01**
Female	26 (34.2)	5 (16.7)	21 (45.65)	
Attributable Factor				
HBV	48 (63.2)	17 (56.7)	31 (67.4)	0.34
HCV	5 (6.6)	1 (3.3)	4 (8.7)	0.36
Alcohol	15 (19.7)	9 (30.0)	6 (13.0)	0.07
Herbal medication	9 (11.8)	4 (13.3)	5 (10.9)	0.75
NAFLD	1 (1.3)	0 (0)	1(2.2)	0.46
Unknown	11 (9.4)	3 (10.0)	8 (17.4)	0.46
Comorbidity				
Yes	5 (11.9)	1 (6.7)	6 (20.0)	0.25
No	37 (88.1)	14 (93.3)	24 (80.0)	

Abbreviations: HBV, hepatitis B virus; HCV, hepatitis C virus; NAFLD, non-alcoholic fatty liver disease.

**Table 4:** Univariable logistic regression analysis of variables associated with in-hospital mortality (outcome) from liver cirrhosis and HCC.

Cirrhosis			
	OR	95% CI	p value
Age	1.00	0.98–1.03	0.57
Gender (Female)	1.5	0.67–3.57	0.32
Duration of admission (days)	0.92	0.85–1.00	0.05
WBC (X10^9^/L)	1.14	1.05–1.23	**<0.001**
Platelet (KU/L)	1.00	1.00–1.00	0.17
INR	1.80	1.06–3.06	**0.03**
Total Bilirubin (mg/dl)	1.01	1.00–1.01	**0.02**
MELD-Na	1.11	1.05–1.19	**<0.001**
Upper GI Bleeding	1.10	0.44–2.69	0.85
Child Pugh Score*			
B	3.55	0.92–13.75	**0.07**
C	8.96	2.35–34.10	**0.001**
Hepatic Encephalopathy**			
Grade 1	3.26	0.86–12.39	0.08
Grade 2	6.79	1.18–39.07	**0.03**
Grade 3	5.43	1.41–20.89	**0.01**
HCC			
	OR	95% CI	p value
Age	1.02	1.00–1.07	0.08
Gender (Female)	4.20	**1.37–12.90**	**0.01**
Duration of admission (days)	0.96	0.89–1.04	0.31
WBC (X10^9^/L)	1.05	0.95–1.15	0.32
INR	1.40	0.79–2.49	0.25
Total Bilirubin (mg/dl)	1.00	1.00–1.01	0.41
MELD-Na	1.04	0.96–1.12	0.37
Upper GI Bleeding	0.35	0.08–1.59	0.17
Hepatic Encephalopathy			
Grade 1	6.75	**1.39–32.87**	**0.02**
Grade 2	1.03	0.06–17.49	0.98

Abbreviations: AFP, alpha-fetoprotein; INR, international normalized ratio; WBC, white blood cells; MELD-Na, Model for End-Stage Liver Disease-Sodium *Base level is Child Pugh A **Base level is no encephalopathy

For the univariate analysis of in-patient mortality among HCC patients, being a female (OR = 4.20, 95% CI: 1.37–12.90) and having hepatic encephalopathy grade 1 (OR = 6.75, 95% CI: 1.39–32.87) were associated with higher odds of death. Variables found to be significant in the univariate models were included in the multivariable model; predictors of in-patient mortality among those with cirrhosis were elevated WBC (OR = 1.14, 95% CI: 1.00–1.30) and MELD-Na score (OR = 1.24, 95% CI: 1.01–1.54) (**Table 5**). Among patients with HCC, female sex (OR = 3.74 95% CI 1.09–12.81) and hepatic encephalopathy grade 1 (OR = 5.66 95% CI 1.10–29.2) were predictors of mortality on multivariable analysis.

**Table 5:** Multivariable logistic regression analysis of predictors of in-patient mortality from liver cirrhosis and HCC.

Cirrhosis			
	OR	95% CI	P value
WBC (X10^9^/L)	1.14	1.00–1.30	**0.04**
INR	0.39	0.11–1.36	0.14
Total Bilirubin (mg/dl)	0.99	0.98–1.00	0.21
MELD-Na	1.24	1.01–1.54	**0.04**
Hepatic encephalopathy			
Grade 1	2.27	0.59–88.31	0.66
Grade 2	9.48	0.52–173.43	0.13
Grade 3	1.59	0.17–14.87	0.69
Child Pugh Score			
B	0.28	0.03–2.44	0.25
C			
HCC			
Gender (Female)	3.74	1.09–12.81	**0.04**
Hepatic Encephalopathy			
Grade 1	5.66	1.10–29.2	**0.04**
Grade 2	0.71	0.04–13.49	0.82

Multivariable model adjusted for age (continuous) gender (male, female), WBC (continuous) MELD-Na (continuous).

Abbreviations: INR, international normalized ratio; WBC, white blood cells; MELD-Na, Model for End-Stage Liver Disease-Sodium

#### Kaplan-Meier survival estimates

**Fig 4:**
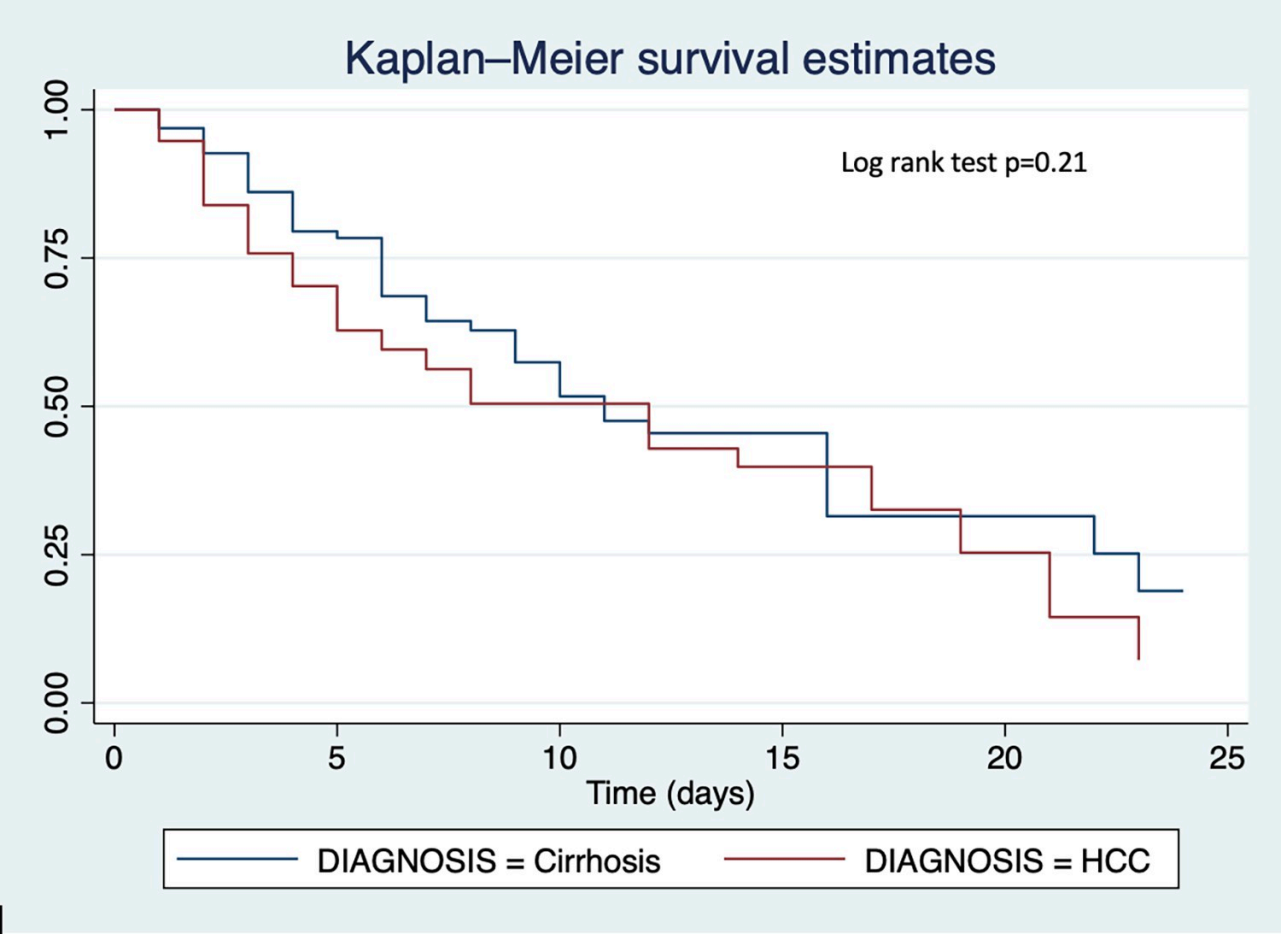
Kaplan-Meier survival curves comparing in-patient survival probabilities between liver cirrhosis patients and HCC patients.

**Fig 5:**
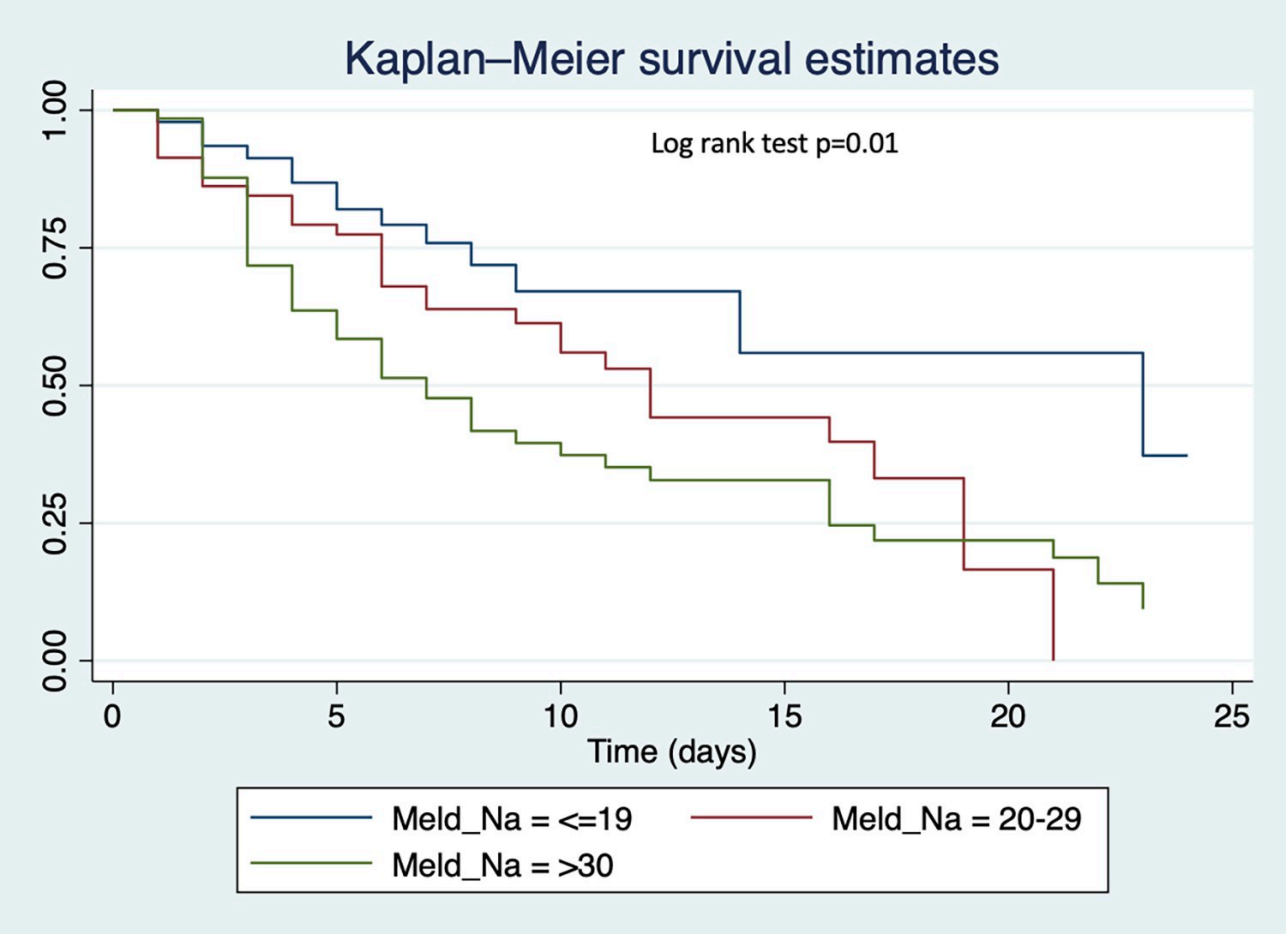
Kaplan-Meier survival curves comparing in-patient survival by MELD-Na score.

**Fig 6:**
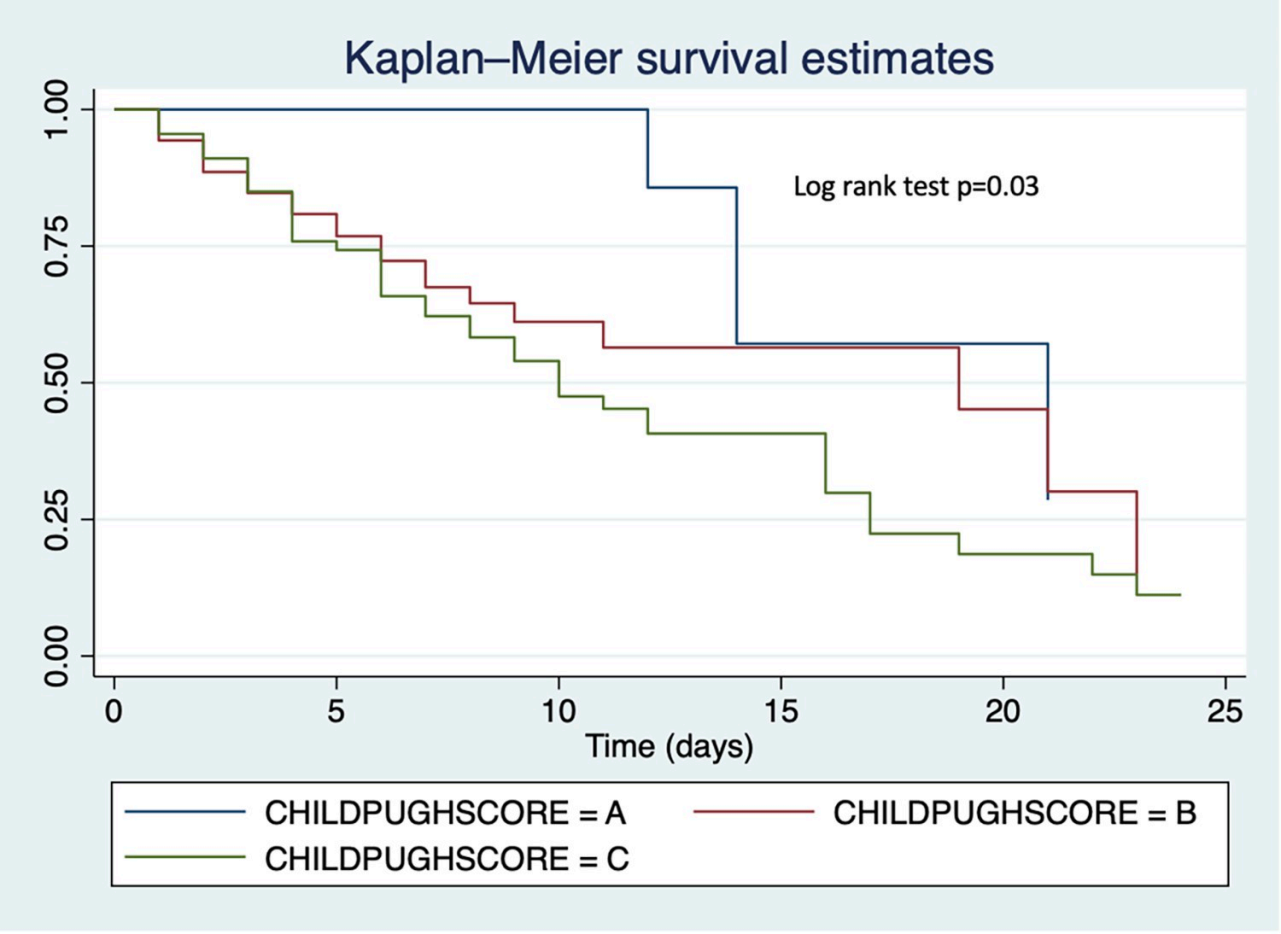
Kaplan-Meier survival *curves* comparing in-patient survival by Child-Pugh score.

Time-to-event analysis was performed using Kaplan-Meier survival estimates comparing in-patient survival probabilities between cirrhosis and HCC, survival probabilities by MELD-Na score (< = 19, 20–29, >30) and by Child-Pugh score (A, B, C). Overall, there was no difference in survival across time between HCC and cirrhosis patients (p = 0.21) (**Fig 4**). However, we found that patients with lower MELD-Na had better survival during admission than those with higher MELD-Na (p = 0.01). Also, patients with Child-Pugh score C had poorer survival as compared to those with Child-Pugh score A or B (p = 0.03) (**Figs 5 and 6**).

## Discussion

In this study, we assessed the proportion of total deaths from liver-related causes, the liver-related mortality associated with liver cirrhosis and hepatocellular carcinoma, and the proportion of these deaths associated with known risk factors in 11 referral hospitals in Ghana. We additionally assessed the in-patient mortality of liver cirrhosis and hepatocellular cancer patients admitted to a tertiary referral centre, and the predictors of mortality in these patients.

The proportion of deaths attributable to liver-related causes was 6.4% across all age-groups and 8.8% in the adult population. Unspecified chronic liver disease accounted for 3.5%, liver cirrhosis for 1.6% and HCC for 1.5% of all deaths among all ages. Modelled estimates from the Institute of Health Metrics and Evaluation (IHME) reported that across all age-groups, liver cirrhosis and other chronic liver diseases were responsible for 2.94% (95% CI 2.27–3.61) of deaths in Ghana in 2019 [18]. The WHO’s modelled estimate of liver cancer cases in Ghana for 2020 was 3,166 [19], or roughly 1.5% of total deaths based on modelled estimates of total number of deaths in the country [20]. This study offers one of the first attempts to report the proportion of deaths from liver-related causes from an observational study in Ghana. Our estimates are close to modelled predictions of disease burden for HCC and cirrhosis, and in part support the accuracy of prediction tools in low-resource settings such as parts of Africa and Asia, where observational studies on mortality may be limited.

We noted that of the liver-related deaths, more than half were classified as chronic liver disease in MCCD registers, without specification of the type of disease. Additionally, over a third of the deaths had no underlying risk factor listed. The possible reasons for these findings are multifactorial. The most probable reason is that liver cirrhosis and liver cancer deaths were reported as chronic liver disease mortality, a common practice among practitioners in Ghana, and that the true proportion on deaths from liver cancer and liver cirrhosis is higher than our reported findings. Errors in reporting cause of death in MCCD registers may occur especially when certificates are signed by non-specialist medical officers [17], and is a possibility here, although we did not collect data on the clinical rank of practitioners certifying deaths. Another reason is the possibility that patients had insufficient test results for practitioners to arrive at a more specific diagnosis, as is common-place in low-resource settings. This may be related to the high cost of care associated with these conditions, which is often a barrier to definitive diagnosis and treatment [15, 21, 22].

This study also described the proportions of liver-related deaths associated with common risk factors such as HBV, HCV, and alcohol. In our study, 69.8% of HCC deaths were associated with HBV infection and 9.4% with HCV, whilst 25% and 13.2% of cirrhosis deaths were associated with HBV and HCV respectively. Modelled estimates suggest that in 2019, 51% of liver cancer deaths were attributable to HBV whilst 14% were attributable to HCV in Ghana [23]. On a global scale, risk factors for HCC-related mortality vary by geographic region with HCV as the predominant risk factor in Northern African countries such as Egypt, and alcohol as the predominant risk factor in Central Europe and North America [24]. The relatively lower burden of HBV-related ESLD in regions such as parts of Europe and North America are related to effective and stringent measures including injection blood safety, infection control strategies, antenatal screening and widespread acceptability of childhood vaccination [25]. Previous studies in Ghana have reported the high proportion of liver cancer and liver cirrhosis cases attributable to HBV infection [4, 15, 16], and this emphasizes the need for improved policies and strategies to reduce the HBV burden in Ghana. Although there is a national policy on viral hepatitis [26, 27], the country has been unable to meet diagnosis and treatment targets for HBV and HCV infection, is yet to attain elimination goals, and has no published financing plan to achieve these aims. Neither HBV nor HCV treatment is free, and patients must pay out-of-pocket [28]. Additionally, the current policies for HBV and HCV care in Ghana rank poorly on summarised policy scores, based on viral hepatitis policy indicators [29]. Furthermore, birth dose vaccination is yet to be implemented in the national immunization plan for new-borns and there is limited viral load and serological profile testing in HBV positive mothers [30, 31].

Another important risk factor in patients with mortality from cirrhosis was alcohol accounting for 33.3% of cirrhosis deaths and 4.1% in those with HCC, and this finding is consistent with others assessing the same in Ghana [32, 33]. The WHO estimated that in 2016, the attributable fraction of alcohol to liver cirrhosis was 39.6% and to liver cancer was 6% in males [34]. Alcohol is an important risk factor, and the need for policies to address poor regulation, marketing, and consumption of alcoholic beverages in the country is what led to a new national alcohol policy in 2017 with the overall aim of reducing alcohol related morbidity and mortality [35].

In this study, we found that close to 1 in 2 patients with cirrhosis or HCC admitted for in-patient care died whilst on admission. Duah et al. found an in-hospital mortality rate of 41.9% in a study among cirrhosis patients admitted to a district hospital in Ghana [14]. It is possible that our mortality rate was higher because this study was conducted in a tertiary referral centre compared to the Duah study, which was conducted in a secondary level referral centre. It is therefore plausible that patients seen at our centre had more advanced or severe disease since patients from secondary referral centres are transferred to higher level canters. Similar studies in West Africa also report high mortality for cirrhosis and HCC cases admitted to medical wards with a study in a tertiary referral centre in South-West Nigeria reporting a very high mortality rate at 82% [36, 37].

In the present study, predictors of poor survival in cirrhosis patients were elevated white cell count, suggestive of infection, and increasing MELD-Na score, whilst hepatic encephalopathy and female sex were predictors of death in HCC patients. Infections have been shown to frequently complicate cirrhosis [38] and were found to precipitate acute-on-chronic liver failure, as well as circulatory and renal system failures, leading to mortality in European and North American Cohorts [39, 40]. Elevated WBC, which may suggest infections, must therefore be thoroughly investigated for early initiation of antibiotic therapy where necessary, and prevention of sequelae such as organ failure, hepatic encephalopathy, and adverse outcomes. In this study, we also found that in-patient survival time was poorer with higher Child-Pugh score. In published studies from Ghana, only Duah et al’s study has investigated predictors of in-hospital mortality among cirrhosis patients. In their paper, and similar to our findings, MELD-Na and hepatic encephalopathy were independent predictors of mortality [14]. As with our findings, MELD-Na score, Child-Pugh score and elevated WBC have also been demonstrated as predictive of mortality in studies in Africa, including Egypt and Ivory Coast [36, 41]. These findings suggest that mortality is higher in patients with more advanced liver disease. Furthermore, precipitants of acute decompensation such as infections, can contribute to mortality. In low resource settings like Ghana where therapeutic and/or curative interventions for cirrhosis and HCC, such as liver transplantation, liver resection and radiofrequency ablation are rarely performed [16], and where best supportive care is often standard care for these patients due to the late stage at presentation [22], it is imperative that treatable causes of decompensation such as infections are promptly identified and managed to prolong life.

The strengths of this study include the multi-centre approach in identifying the burden of liver-related mortality and the proportion of liver cirrhosis and HCC deaths associated with known risk factors. Additionally, as one of the few studies to investigate predictors of liver cirrhosis and liver cancer mortality in a Ghanaian cohort, it contributes to the body of local literature necessary to improve clinical care. Limitations of this study include the use of secondary data, and the resultant challenge of missing or unavailable data despite data validation, on key variables and gross categorization of chronic liver disease without details on the subtype as highlighted above. Also, the close to half of chronic liver disease deaths were certified with no antecedent cause, and this may reflect uncertainty from clinicians in the diagnosis or limitations in arriving at a definitive diagnosis before death. Additionally, we did not assess quality of death certification by medical officers, therefore we cannot speak to the quality of reported data in the MCCD registers. It is however important to note that WHO and other modelled estimates are often based on data reported in the DHIMS, derived from MCCD registers in the country. Furthermore, we collected data from government health facilities, where the socioeconomic status is generally lower and risk factors and outcomes may potentially differ from private or more affluent healthcare facilities in the country.

### Conclusion

Sustainable and focused medium- and long-term strategies are required to overcome the burden of disease and to reduce mortality from liver cirrhosis and liver cancer in Ghana. These may include tactics such as creation of surveillance systems and registries that monitor patients who are at risk of developing complications of chronic liver disease, development of cheaper and non-invasive markers for the diagnosis of cirrhosis and HCC, and incorporation of viral hepatitis, liver cirrhosis and HCC diagnosis and treatment into government-financed care. Additionally, there is a need for policy development based on robust and reliable nationwide data, which in turn requires new and improved reporting systems, and training of healthcare personnel using specific and internationally acceptable case definitions.
